# Could aspirin be a lifesaver for prostate cancer patients in prostate cancer-specific mortality?: an update systematic review and meta-analysis

**DOI:** 10.1186/s12885-019-6415-5

**Published:** 2019-12-05

**Authors:** Jiatong Zhou, Shuai Xia, Tao Li, Ranlu Liu

**Affiliations:** Department of Urology, The Second Hospital of Tianjin Medical University, No. 23, Pingjiang Road, Hexi District, Tianjin, 300211 China

**Keywords:** Aspirin, Prognosis, Prostate cancer, Meta-analysis

## Abstract

**Background:**

Currently, clinical studies on the prognosis of prostate cancer (PC) taking aspirin were developing, but the precise mechanism of aspirin on tumor cells was still unclear. In addition, the conclusion that aspirin can improve the prognosis of PC patients continues to be controversial. Therefore, we collected comprehensive literatures and performed our study to explore the prognostic effect of aspirin on PC.

**Methods:**

A comprehensive literature search was performed in April 2019 based on PUBMED. EMBASE. Hazard Ratio (HR) as well as its 95% confidence interval (CIs) for prostate cancer specific mortality (PCSM) was extracted from eligible studies.

**Result:**

A total of 10 eligible articles were used in our study. The pooled results showed that PC patients who used aspirin or taking aspirin did not have lower PCSM than those who had not used (HR =0.89, 95% CI: 0.73–1.08, *P*>0.05). In subgroup analysis, we found that taking aspirin before diagnosis of prostate cancer and taking aspirin after diagnosis of prostate cancer did not have significant association with PCSM. (pre-diagnostic use, HR = 0.88, 95% CI: 0.72–1.06; post-diagnosis use, HR = 0.88, 95% CI: 0.67–1.17). In addition, we found no significant association between aspirin use or its duration and the risk of PCSM. Another important result demonstrated that aspirin use was not associated with risk of PSCM in either high risk (T ≥ 3 and/or Gleason score ≥ 8) or low risk PC patients(low-risk PC, HR = 1.05, 95% CI: 0.81–1.35; high-risk PC, HR = 0.97, 95% CI: 0.75–1.24).

**Conclusion:**

Our results demonstrated that there was no significant association between aspirin use and the risk of PCSM. At the same time, the dosage and duration of aspirin use had no statistical influence on the risk of PCSM in high/low risk PC. Further studies are needed to confirm the findings.

## Background

Aspirin use could effectively fight inflammation and has a good anti-inflammatory effect, which can inhibit tumor cell growth and inhibit tumor progression [1]. Some preclinically basic studies had found some possible anti-tumor mechanisms of aspirin in prostate cancer (PC). The main mechanism of aspirin is inhibition of cyclooxygenase 2. PC cells overexpressed cyclooxygenase 1/2 (COX-1/2). Thereby, aspirin inhibited tumor cell growth and distant metastasis [2, 3]. Downer et al. suggested that regular aspirin use was associated with a lower risk of lethal PC [4]. While Assayag et al. observed that post-diagnostic use of aspirin was not associated with a risk of prostate cancer outcomes [5]. So, there was still controversy between different studies.

Due to the controversy still existed between different researches, we performed this meta-analysis to discuss whether aspirin could reduce the risk of prostate cancer-specific mortality (PCSM).

## Methods

### Literature search

**Fig. 1:**
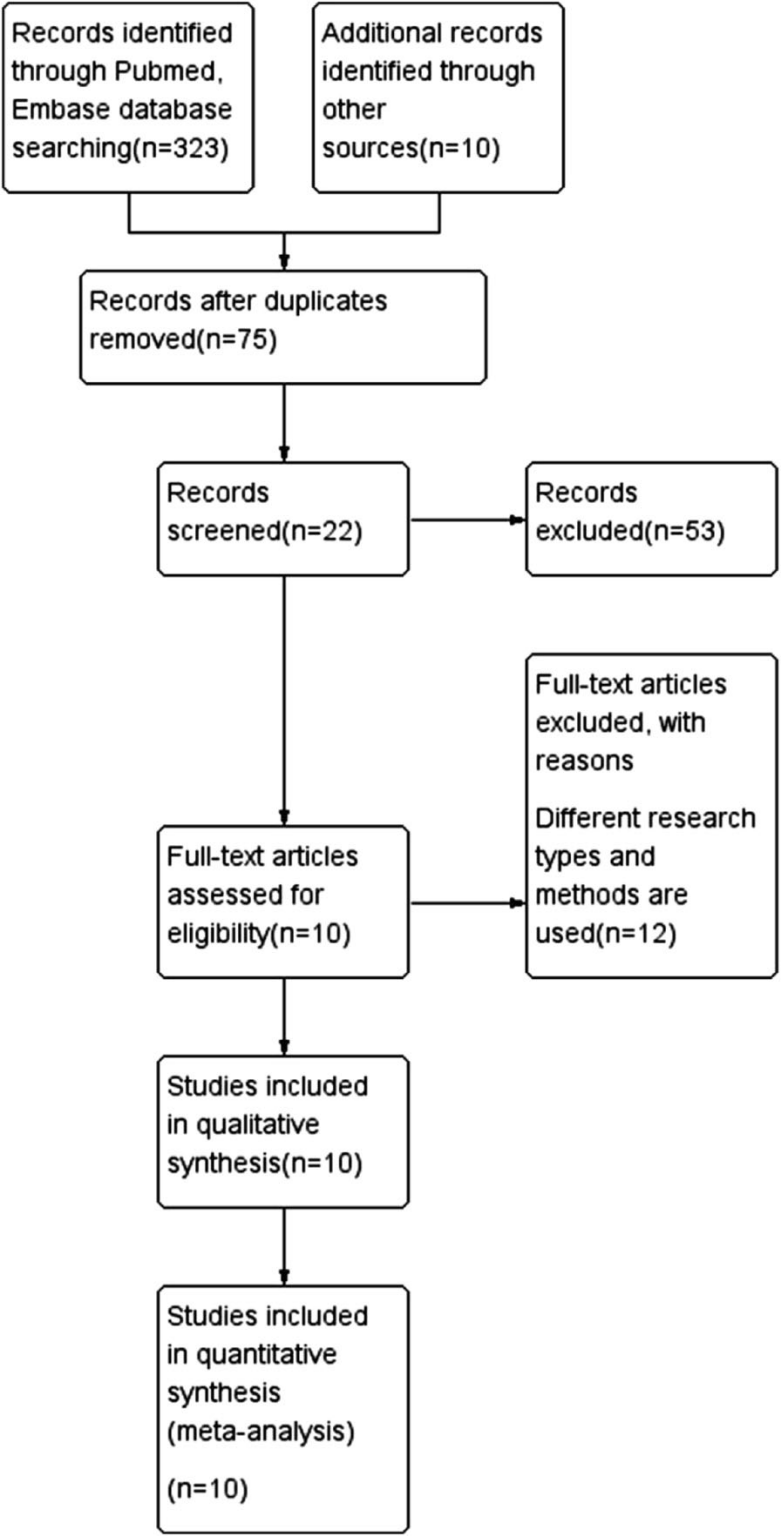
Flow diagram of included studies for this meta-analysis

We conducted the literature research in April 30th 2019 and chose the related study from PubMed, Web of Science databases, Embase with the following keywords: (“prostate cancer” or “prostate tumor”), (“aspirin” or “acetylsalicylic acid”), (“prostate cancer-specific mortality” or “PCSM”). In the process of searching for literature, we did not limit the language and publication time of publication. The flow diagram was demonstrated in Fig. 1. The present systematic review and meta-analysis were designed, performed, and reported in accordance with the standards of quality for reporting meta-analysis [6].

### Inclusion criteria and exclusion criteria

Researches meeting the following requirements were included in our study: the important concern was aspirin use; the reslut of this concern was PCSM, study design was prospective/retrospective cohort study or randomized controlled trial. We get the final HR and its CI from the data extracted in the included articles.

Exclusion requirements included the following: (1) reviews, letters, comments, and animal studies; (2) duplicate publications; (3) studies without complete data.

### Quality assessment

The Newcastle–Ottawa Scale (NOS) [7] scoring system was used by us to evaluate the quality of literature we have included by two independent reviewers (Tao L, Shuai X). If a study with ≥7 points which could be considered as a high-quality study [6].

### Data extraction

Same 2 reviewers (Tao L, Shuai X) selected data and deliberated any divergences. The information which extracted from the included literatures: first author’s surname, the country, publication year, type of study design, number of cases and controls, HR value and its 95% CIs.

### Statistical methods

For studies that reported HR and its 95% CIs for PCSM, we concluded these risk estimates within each study. Heterogeneity across included studies was assessed with the Cochran Q test [8]. Galbraith plot [9] was used to find the studies that resulted in the main heterogeneity. Pooled HRs were calculated using random-effects models of DerSimonian and Laird.

We reconducted this meta-analysis after omitting each study in sensitivity analysis. Potential publication bias was evaluated with Begg’s test [10], and *p* < 0.05 was considered as statistical difference. Statistical analyses were completed with Stata SE12.0.

## Results

### Study search and characteristics

The detailed process of literature search is shown in Fig. 1. A total of 10 eligible studies were finally included in the present meta-analysis. These studies were carried out in the following geographical regions: USA (*n* = 5), Canada(*n* = 2), UK(*n* = 1), Ireland (*n* = 1) and Finland(*n* = 1). There were 10 cohort studies [4, 5, 11–18], which were published from 2008 until 2018. Information on exposure (aspirin) was collected by clinical detection. Study outcome (PCSM) was proved in all included studies. Quality scores evaluated by the Newcastle–Ottawa Scale (NOS) ranged from 7 to 8. The main characteristics of all included studies have been summarized in Table 1.

**Table 1:** Characteristics of studies included in the meta-analysis

Study	Country	Year	PC case	Died from PC or develop lethal PC	Type of study	Drugs studied	Treatment	HR, 95% CI
Veitonm€aki	Finland	2015	6537	617	cohort	aspirin/NSAID	RP, RT, ADT or observe NSAID	1.067(0.774–1.471)
Downer	USA	2017	3277	492	cohort	aspirin	RP, RT	0.645(0.488–0.852)
Flahavan	Ireland	2014	2936	276	cohort	aspirin	RP, RT, ADT	0.88(0.67–1.15)
Jacobs	USA	2014	8427	441	cohort	aspirin	RP, RT, ADT	0.946(0.788–1.135)
Zhou	USA	2017	26,890	975	cohort	aspirin/NSAID	RP, RT, ADT	0.976(0.874–1.09)
Assayag	Canada	2015	11,779	1793	cohort	aspirin	RP, RT, ADT	1.46(1.29–1.65)
Hurwitz	USA	2018	817	90	cohort	aspirin/NSAID	NR	0.59(0.36–0.96)
Choe	USA	2012	5955	193	cohort	aspirin	RP, RT	0.43(0.21–0.87)
Rothwell	UK	2011	Nr	Nr	cohort	aspirin	NR	0.52(0.2–1.34)
Stock	Canada	2008	1619	453	cohort	NSAID	RP, RT	1.03(0.79–1.34)

*PC* prostate cancer, *HR* hazard ratio, *CI* confidence interval, *NR* not report, *RP* radical prostatectomy, *RT* radiotherapy, *ADT* antiandrogen therapy, *NSAID* nonsteroidal anti-inflammatory drug

### Association between aspirin and PCSM of PC patients

**Fig. 2:**
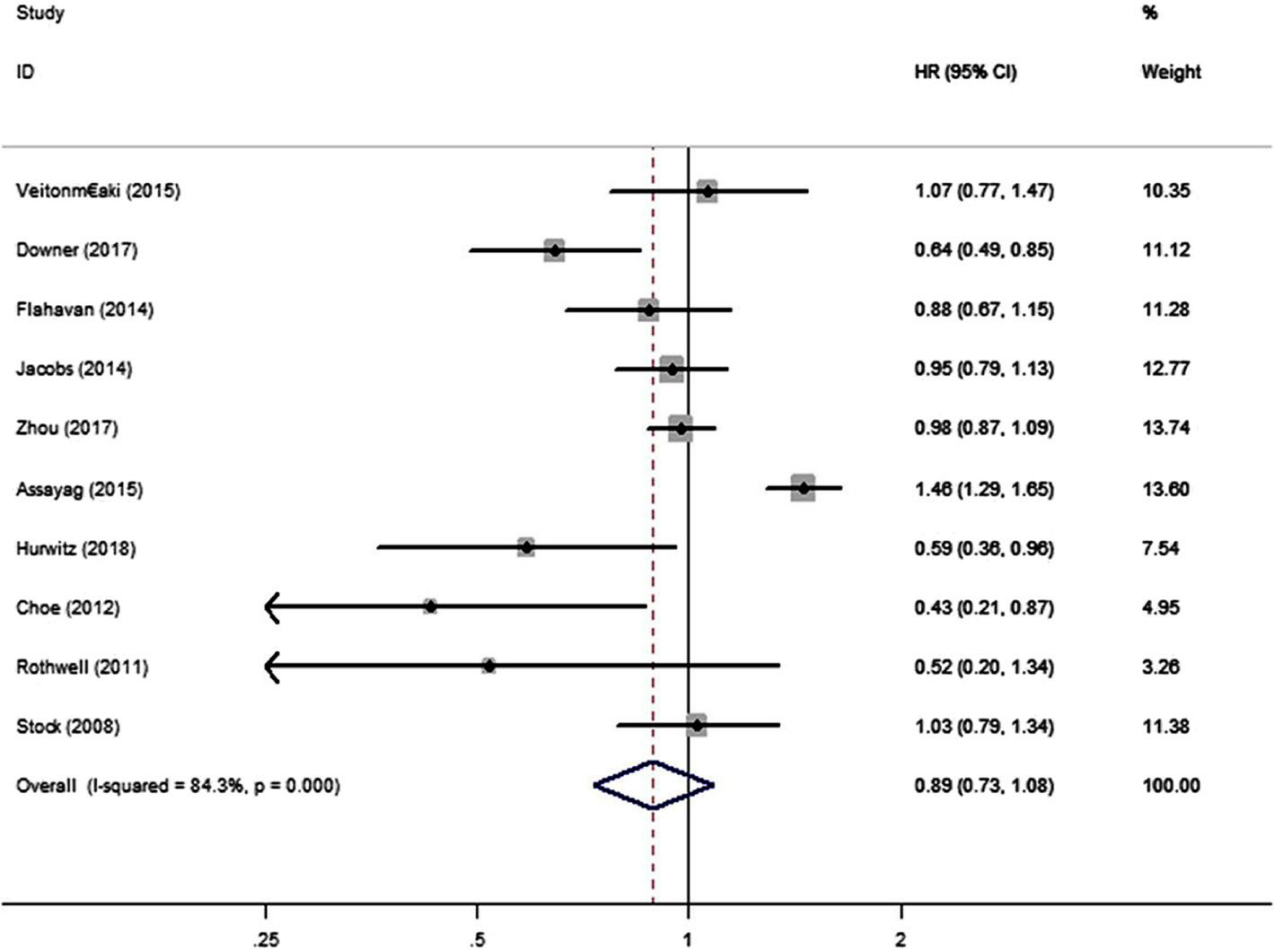
Association between aspirin use and risk of PCSM. HR, hazard ratio; CI, confidence interval; PCSM, prostate cancer-specific mortality

**Fig. 3:**
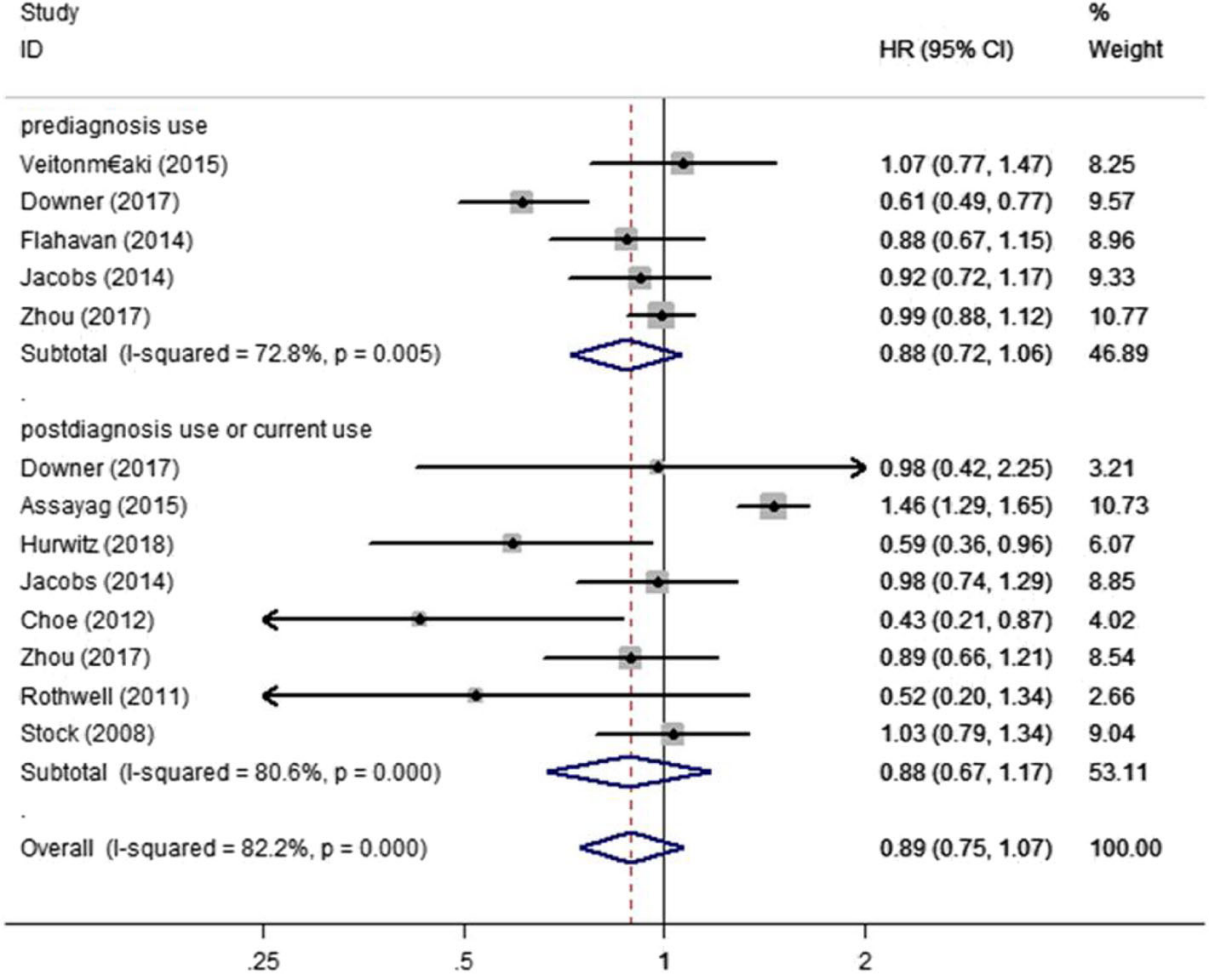
Association between aspirin use and risk of PCSM stratified by timeframe of use. HR, hazard ratio; CI, confidence interval; PCSM, prostate cancer-specific mortality

**Fig. 4:**
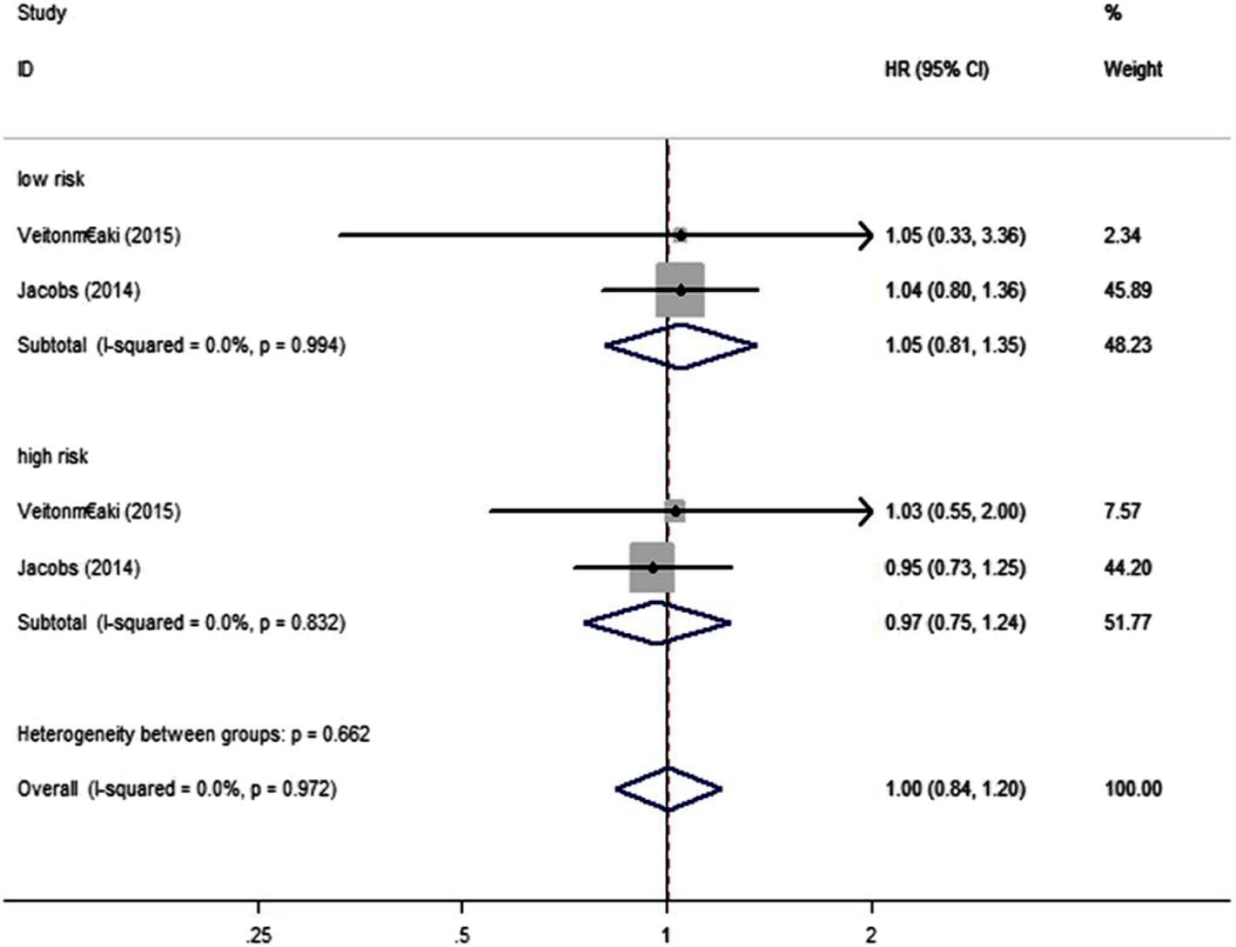
Association between aspirin use and risk of PCSM stratified by disease severity. HR, hazard ratio; CI, confidence interval; PCSM, prostate cancer-specific mortality

**Fig. 5:**
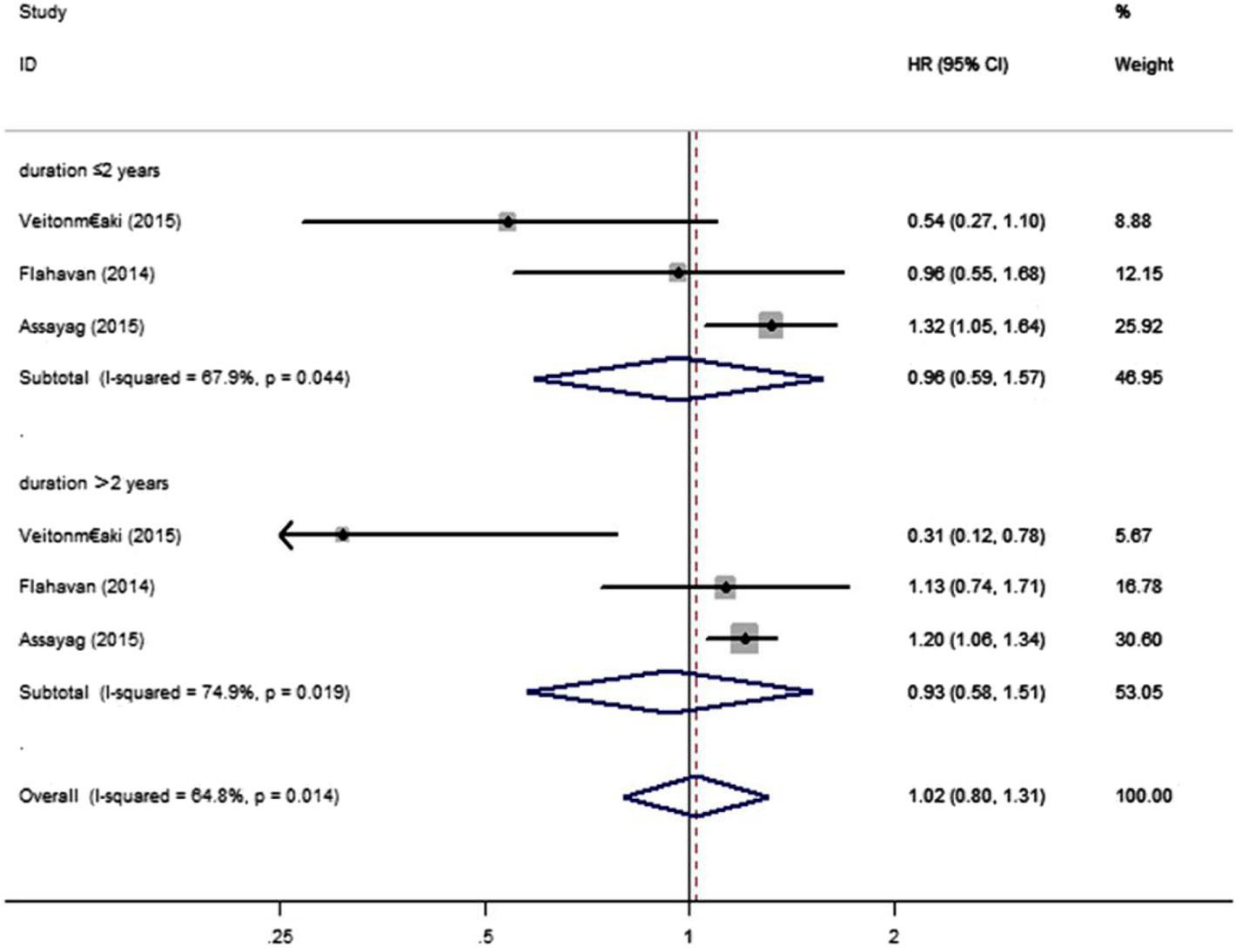
Association between duration of aspirin use and risk of PCSM. HR, hazard ratio; CI, confidence interval; PCSM, prostate cancer-specific mortality

**Fig. 6:**
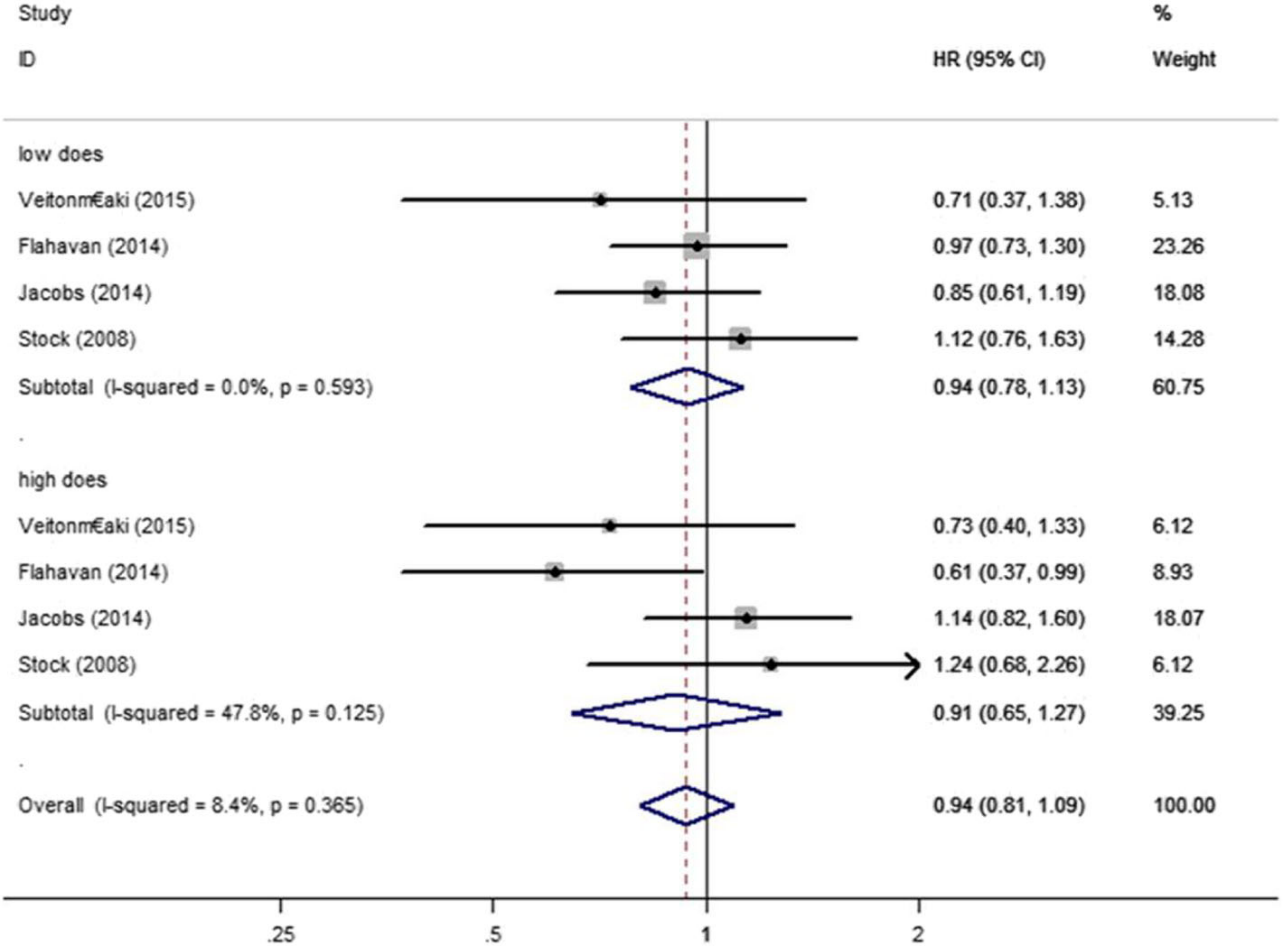
Association between duration of aspirin use and risk of PCSM stratified by dosage of aspirin. HR, hazard ratio; CI, confidence interval; PCSM, prostate cancer-specific mortality

We examined the relationship between aspirin and PCSM of PC patients using data from 10 studies [4, 5, 11–18]. We discovered that aspirin was not significantly associated with risk of PSCM (HR = 0.89, 95% CI: 0.73–1.08, *P*>0.05) (Fig. 2). Through different subgroup analysis, we found that there was no significant difference in PCSM either pre-diagnostic use or post-diagnosis use (pre-diagnostic use, HR = 0.88, 95% CI: 0.72–1.06, I^2^ = 72.8%; post-diagnosis use, HR = 0.88, 95% CI: 0.67–1.17, I^2^ = 80.6%) (Fig. 3). And also, aspirin was not significantly associated with risk of PSCM in patients with high or low risk PC. Low risk PC was defined as Topography(T) ≤ 2 or Gleason score ≤ 7, high risk PC was defined Topography(T) ≥ 3, Gleason score ≥ 8 or distant metastasis. (low risk PC, HR = 1.05, 95% CI: 0.81–1.35, I^2^ = 0.00%; high risk PC, HR = 0.97, 95% CI: 0.75–1.24, I^2^ = 0.00%) (Fig. 4). There was no significant association between aspirin duration and PCSM (duration>2 years, HR = 0.96, 95% CI:0.59–1.57, I^2^ = 67.9%; duration≤2 years, HR = 0.93, 95% CI:0.58–1.51, I^2^ = 74.9%) (Fig. 5). In terms of drug dose (low dose ≤75 mg, high dose > 75 mg), we discovered that aspirin dose had also no significant association with PCSM (low dose, HR = 0.94, 95% CI:0.78–1.13, I^2^ = 0.00%; high dose, HR = 0.94, 95% CI:0.75–1.19, I^2^ = 8.4%) (Fig. 6).

### Sensitivity analysis and publication bias

**Fig. 7:**
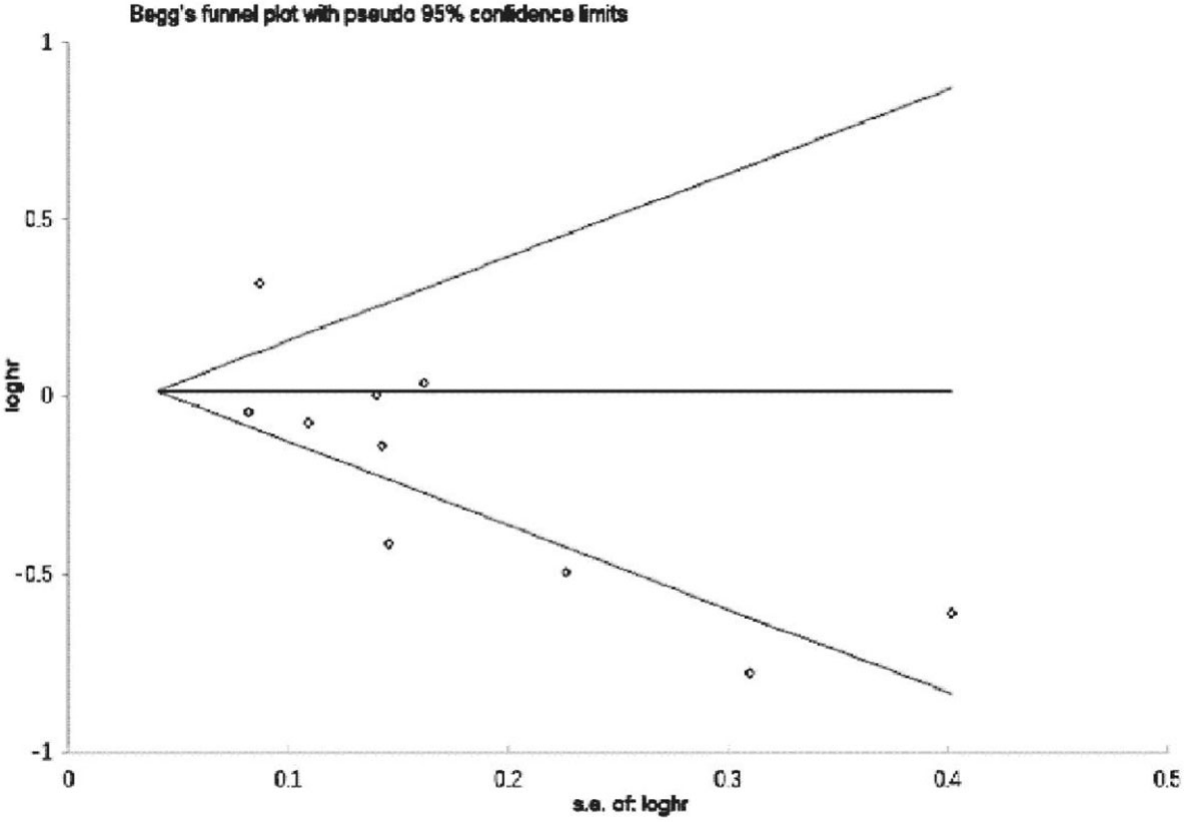
Funnel plots based on aspirin use and PCSM (Begg’s test)

**Fig. 8:**
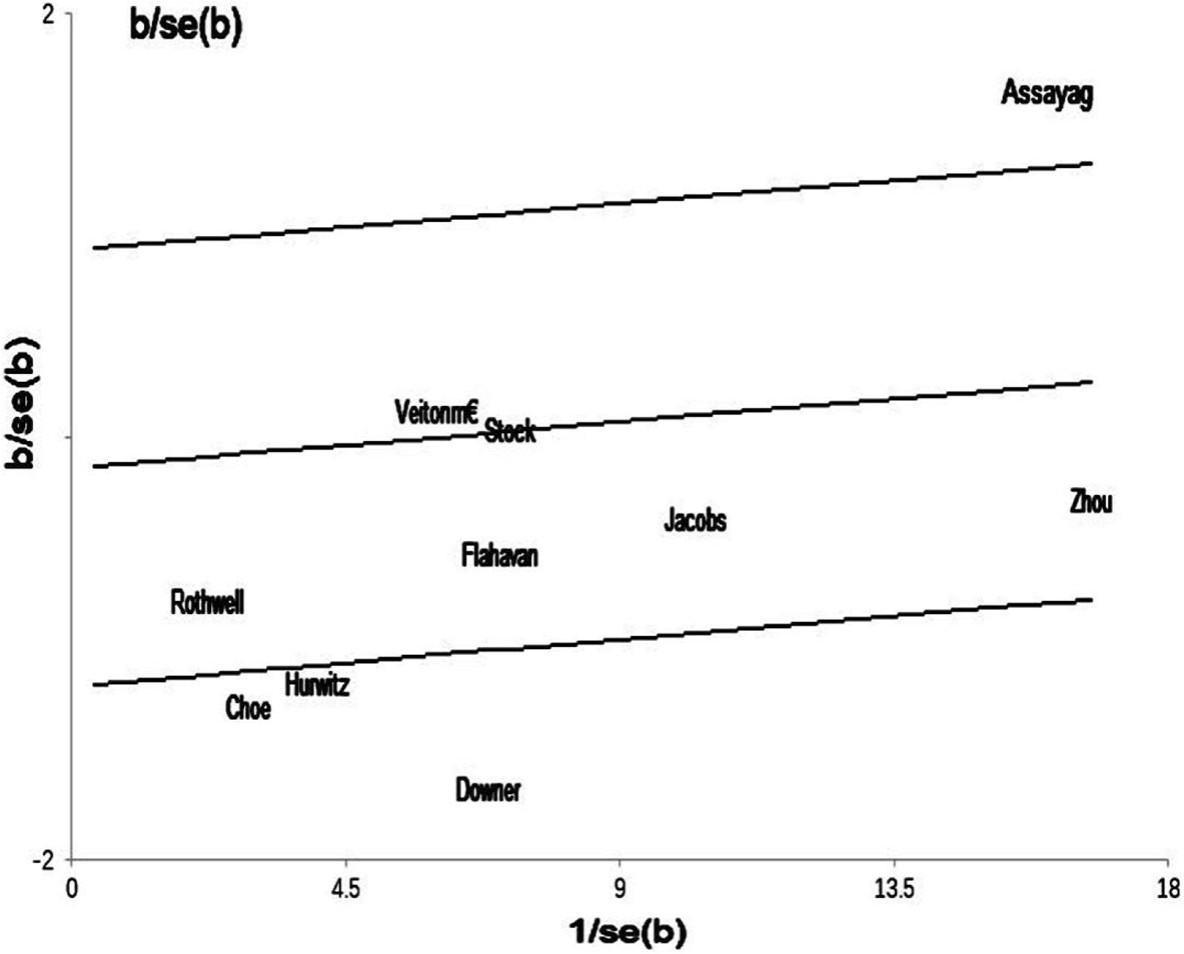
Galbraith plots based on aspirin use and PCSM

Sensitivity analysis confirmed that no individual research influenced the pooled outcome on HR. There was no publication bias in our study by the Begg’s funnel plot (*p* = 0.238) (Fig. 7). And we performed Galbraith plot and found that there was heterogeneity in the literatures included in our study (Fig. 8).

## Discussion

Our study assessed the association between aspirin use and PCSM from 10 clinics researches [4, 5, 11–18]. With the use of aspirin in clinical practice, many studies have focused on the role of aspirin in anti-cancer treatment, but the results continued to be controversial. We had concentrated the association between aspirin use and the risk of PCSM and found that aspirin had no significant connection on prolonging survival time in PC patients. According to the overall results, we conducted subgroup analysis divided by different factors, including medication duration, dosage, tumor grade, and whether use aspirin after diagnosis of PC or before diagnosis. We discovered that aspirin did not reduce the risk of PCSM in these subgroups. As we all know, the parthenogenesis of tumors is unclear. At present, it was believed that genetic change may be an important mechanism leading to the progression of tumor. Inflammation is also considered to be a meaningful factor. Cyclooxygenase-2 (COX-2), an irreducible enzyme, is overexpressed in PC tissue [19]. In another study, they also confirmed that cyclooxygenase played a major role in prostate tumor cells [20]. After taking into account these reports, we hypothesized that inhibiting inflammatory cytokines might have a protective effect on tumor patients. Aspirin is successful in inhibiting COX-2 and it might reduce the risk of PCSM, improving the prognosis of PC patients. Choe et al. reported that aspirin was linked to the risk of PCSM in men treated with radical prostatectomy (RP) or radiotherapy (RT) for PC(P<0.05) [13]. Subsequently, Downer et al. demonstrated in 2017 that regular use of aspirin reduced the risk of advanced PC (defined as tumor metastases to bones or other organs or death if the cause of death was PC) [4]. These studies supported the protective influence of aspirin on PC patients and delayed the progression of PC. Hurwitz et al. recently discovered that aspirin use was inversely associated with the risk of PCSM [12]. However, they found that aspirin did not effectively reduce the incidence of PC. Liu et al. in 2014 [6] performed a meta-analysis, which gave us the conclusion that aspirin use was significantly associated with incidence risk of PC, and in subgroup analysis, aspirin had a statistical association with advenced PC. Depending on their results, aspirin use was inversely related to the risk of PCSM. Their results are in agreement with the findings of the above study, but contradicting our final conclusion, we did not suggest that aspirin could play a particularly significant role in the risk of PCSM. Among the articles we had included, Assayag et al. in 2015 reported post-diagnostic aspirin use was linked to an increased risk of PCSM [5]. Veitonmäki et al. observed that the risk of PC death associated with NSAID (non-steroidal anti-inflammatory drugs) usage regarding whether diagnosis of PC [13]. However, according to their report, we discovered that aspirin was not significantly associated with the risk of PCSM. Their results could also give a clinical indication that aspirin might not improve the prognosis of PC patients, but other NSAIDs might affect the growth of PC cells from a mechanism different from aspirin. Other NSAIDs might promote tumor progression and increase the risk of PC death. Cumulative use of NSAID and medication intensity may increase risk PC death proposed by Veitonmäki et al. [18]. Flahavan et al. suggested there was no association between pro -diagnostic aspirin use and the risk of PCSM for localized PC [4], but current high doses (> 75 mg) of aspirin could reduce PCSM. From their study, treatment of PC combined with high-dose aspirin may decrease the risk of PCSM. However, it should be pointed out that high doses of aspirin use can cause spontaneous bleeding. Jacobs et al. also thought neither pre-diagnosis nor post-diagnosis daily aspirin use was significantly associated with the risk of PCSM [14]. In patients with high risk PC, daily aspirin use significantly decreased the risk of PCSM. In analyzing low-risk PC death, they found that daily high-dose PC patients did not have lower PCSM than those uses daily lower dose. This seemed to contradict Flahavan’s conclusions. The cause of this contradiction had not been elucidated and may be linked to the underlying disease, race, region and other factors of the patient. Zhou et al. collected two large US prospective cohorts in 2017 [15]. From the analysis of these two studies, they observed pro- and post-diagnostic aspirin or non-aspirin NSAID use was not statistically significantly related with the risk of PCSM, but pro- and post-diagnostic aspirin could reduce all-cause mortality. Therefore, authors suggested that aspirin could prolong the over survival (OS) of PC patients, but could not statistically reduce the risk of PCSM. Since most PC patients are elderly patients, many patients could have cardiovascular diseases, including coronary heart disease, cerebral congestion, myocardial infarction, etc., requiring long-term aspirin antithrombotic therapy, which might reduce mortality. Whether aspirin could be an effective drug for inhibiting the growth of PC cells and improving the prognosis of patients has not yet to be identified. Jacobs et al. in 2014 reviewed 74 high-risk PC patients treated with radiation therapy and they found that aspirin reduced the distant metastasis rate of elevated high risk PC and played a major role in prolonging OS among PC patients [21]. Regardless of the fact that we did not find that taking aspirin can effectively reduce the risk of PCSM in our results, the overall HR trend is still biased to less than 1, and aspirin use may be a protective factor, which was conducive to the survival of PC patients. In the latest review results, Fan et al. collected related researches and supposed the view that there was no association between aspirin exposure and the risk of PCSM [22], and they also found no significant reduction in all-cause mortality in population using the highest dose of aspirin [18]. The authors’ views were consistent with the overall results we obtained, but we did not know more about the effects of aspirin use on high-risk or low-risk PC death. Some clinical studies agreed that aspirin could prolong over survival. This conclusion might promote aspirin to become a hope for PC patients.

### Limitations

Several limitations should be acknowledged in our study. First, the final result may be affected by ethnicity, region, and environment of the study population. Second, the treatment of PC was different, and the underlying diseases of each individual are different, which would affect the therapeutic effect of aspirin use. Third, due to the limited number of researches included in our study, we still need to validate the accuracy of our conclusions through further researches.

## Conclusion

According to our research, aspirin use did not achieve a statistically significant reduction in PCSM of PC, but according to related studies, aspirin could reduce all-cause mortality. Therefore, it was demonstrated that aspirin had a protective effect on cancer patients, but may not inhibit the growth of tumor cells. We still needed a lot of relevant researches to explore the specific link between aspirin and PC survive.

## Data Availability

All data generated or analyzed during this study are included in this published article.

## Abbreviations

HR: Hazard Ratio
NOS: Newcastle–Ottawa Scale
PC: prostate cancer
PCSM: prostate cancer-specific mortality

## References

[1] Chan TA (2002). Nonsteroidal anti-inflammatory drugs, apoptosis, and colon-cancer chemoprevention. Lancet Oncol.

[2] Liu XH (2000). Inhibition of cyclooxygenase-2 suppresses angiogenesis and the growth of prostate cancer in vivo. J Urol.

[3] Nash GF (2002). Platelets and cancer. Lancet Oncol.

[4] Downer MK (2017). Regular aspirin use and the risk of lethal prostate cancer in the physicians’ health study. Eur Urol.

[5] Assayag J, Pollak MN, Azoulay L (2015). The use of aspirin and the risk of mortality in patients with prostate cancer. J Urol.

[6] Liu Y (2014). Effect of aspirin and other non-steroidal anti-inflammatory drugs on prostate cancer incidence and mortality: a systematic review and meta-analysis. BMC Med.

[7] Stang A (2010). Critical evaluation of the Newcastle-Ottawa scale for the assessment of the quality of nonrandomized studies in meta-analyses [J]. Eur J Epidemiol.

[8] Higgins JPT, Thompson SG (2002). Quantifying heterogeneity in a meta-analysis. Stat Med.

[9] Bax L (2009). More than numbers: the power of graphs in meta-analysis. Am J Epidemiol.

[10] Seagroatt V, Stratton I (1997). Bias in meta-analysis detected by a simple, graphical test. Test had 10% false positive rate. BMJ.

[11] Flahavan EM (2014). A cohort study investigating aspirin use and survival in men with prostate cancer. Ann Oncol.

[12] Hurwitz LM (2019). Aspirin and non-aspirin NSAID use and prostate cancer incidence, mortality, and case fatality in the atherosclerosis risk in communities study. Cancer Epidemiol Biomark Prev.

[13] Choe KS (2012). Aspirin use and the risk of prostate cancer mortality in men treated with prostatectomy or radiotherapy. J Clin Oncol.

[14] Jacobs EJ (2014). Daily aspirin use and prostate cancer-specific mortality in a large cohort of men with nonmetastatic prostate cancer. J Clin Oncol Off J Am Soc Clin Oncol.

[15] Zhou CK (2017). Do aspirin and other NSAIDs confer a survival benefit in men diagnosed with prostate cancer? A pooled analysis of NIH-AARP and PLCO cohorts. Cancer Prev Res.

[16] Rothwell PM (2010). Effect of daily aspirin on long-term risk of death due to cancer: analysis of individual patient data from randomised trials. Mdica.

[17] Stock DC (2008). Effects of non-selective non-steroidal anti-inflammatory drugs on the aggressiveness of prostate cancer. Prostate.

[18] Veitonmäki T (2015). Use of non-steroidal anti-inflammatory drugs and prostate cancer survival in the finnish prostate cancer screening trial. Prostate.

[19] Gupta S (2015). Over-expression of cyclooxygenase-2 in human prostate adenocarcinoma. Prostate.

[20] Yoshimura R (2015). Expression of cyclooxygenase-2 in prostate carcinoma. Cancer.

[21] Jacobs CD (2014). Aspirin improves outcome in high risk prostate cancer patients treated with radiation therapy. Cancer Biol Ther.

[22] Fan LL (2019). Aspirin exposure and mortality risk among prostate cancer patients: a systematic review and meta-analysis. Biomed Res Int.

